# Hypoganglionosis in pregnancy: a case report

**DOI:** 10.1186/1752-1947-6-297

**Published:** 2012-09-13

**Authors:** Ana Figueiredo, Isabel Martins, Fátima Palma, Maria José Alves, Carlos de Barros

**Affiliations:** 1 Maternidade Dr. Alfredo da Costa, Rua do Viriato, Lisbon, 1069-089, Portugal

## Abstract

**Introduction:**

We report a very rare case of isolated hypoganglionosis first diagnosed during early pregnancy, which should be discussed from an obstetric and a gastroenterological point of view.

**Case presentation:**

A pregnant 18-year-old Caucasian woman presented at twelve weeks of gestation with lower abdominal pain, mild constipation and a large abdominal mass. Abdominal and pelvic magnetic resonance imaging demonstrated a megarectum and megasigmoid, and our patient was managed with medical therapy during her pregnancy, which occurred without major incidents. At the onset of labor, a fecaloma obstructing the pelvic outlet was detected, which required manual disimpaction. However, during the procedure a sudden continuous fetal bradycardia was detected. An emergency Cesarean section was performed but the fetus suffered hypoxic ischemic encephalopathy. One year after the delivery, our patient underwent a sigmoid resection. A histopathological analysis revealed a reduction of nerve cells in the myenteric and submucous plexus, suggesting hypoganglionosis.

**Conclusion:**

Although there are some reports of pregnancies complicated by megacolon, they are too few and too old to delineate guidelines for clinical orientation. In our article, we discuss several issues regarding the management of these rare intestinal innervation disorders during pregnancy that we believe will enhance their obstetric and gastroenterological management during pregnancy.

## Introduction

One of the effects of progesterone during pregnancy is the diminution in tone of smooth muscle, recognized as the probable cause of the dilatation of the ureters, varicose veins and constipation [1]. As the pregnancy progresses and the uterus expands to fill the pelvis and abdomen, constipation may become a significant issue in prenatal care [2].

What consequences does the atony of an already dilated colon have? And how should a megacolon be managed during pregnancy? Although there are some reports of pregnancies complicated by megacolon [1-4], they are too few and too old (most of them were published more than 20 years ago) to delineate guidelines for clinical orientation.

We present the case of an initial diagnosis of megacolon during pregnancy, which was then revealed to be a case of isolated hypoganglionosis (IH).

## Case presentation

An 18-year-old primiparous Caucasian woman attended our hospital complaining of lower abdominal pain, chest pain, breathlessness and easy tiredness over the past three weeks. She was pregnant although she could not state precisely when her last menstrual period had taken place and had not yet begun her prenatal care. She denied complaints of fever, nausea, vomiting, heartburn, urinary symptoms and vaginal bleeding or discharge. However, she had a history of mild chronic constipation since childhood, with sporadic use of laxatives, which had never been investigated.

Upon physical examination, our patient was hydrated, well-nourished, had a normal blood pressure and heart rate, and had no respiratory distress. On gynecological examination, it was impossible to insert the speculum and bimanual palpation revealed a large abdominal mass of hard consistency extending from her right iliac fossa to her right upper quadrant, apparently displacing our patient’s uterus to the left. A large quantity of impacted stools was found within her rectum and anal verge.

A pelvic ultrasound revealed a single *in uterus* pregnancy of twelve weeks and six days (by biometric measures) and a solid dense tumor occupying the right quadrants of her abdomen, which made it difficult to correctly assess her ovaries.

**Figure 1:**
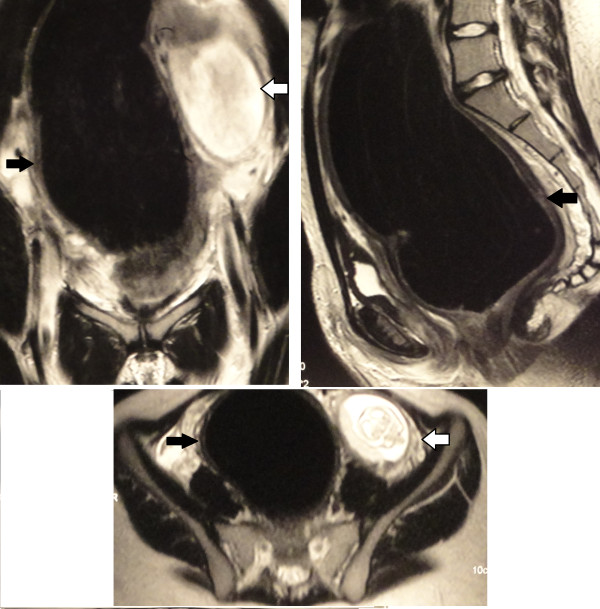
Magnetic resonance images showing megarectum and megasigmoid (black arrows) displacing the pregnant uterus to the left (open arrow).

To classify the nature of the tumor, we performed abdominal and pelvic magnetic resonance imaging (MRI) because this is considered to be a safe imaging study with regards fetal exposure to radiation. The MRI demonstrated a large abdominal and pelvic mass consistent with a megarectum and megasigmoid (Figures 1), with fecal impaction, which exerted a mass effect over the adjacent structures, including her uterus. Her kidneys were normal and there was no free liquid in her abdominal cavity.

Routine laboratory blood tests were unremarkable. Her thyroid function was normal, and her results were negative for toxoplasma, rubella virus, cytomegalovirus and herpes simplex virus infection and for tumor markers (α-fetal protein, carbohydrate antigen 125 and carcinoembryonic antigen).

After discussing the case in a multidisciplinary meeting (with Gastroenterology, General Surgery and Radiology) and considering the mild nature of our patient’s constipation, we decided to manage her megacolon during pregnancy with medical treatment alone. An osmotic laxative (magnesium hydroxide) was begun. A monthly prenatal appointment was planned to evaluate maternal complaints and weight gain as well as to monitor fetal development.

During pregnancy, our patient maintained mild constipation interspersed with short periods of diarrhea. She was admitted to our hospital in the 28^th^ week for profuse diarrhea with no clinical or laboratory signs of infection and was discharged 24 hours later. The remainder of her pregnancy occurred with no other gastroenterological or obstetric incidents, including obstructive symptoms, with normal maternal weight gain and fetal development.

At 39 weeks of gestation, our patient returned to our hospital complaining of colicky abdominal pain. A nonstress test did not reveal any signs of fetal distress and showed an irregular pattern of uterine contractions. On vaginal examination, the fetal head was engaged but it was not possible to evaluate the uterine cervix because of a solid mass of impacted stools that was obstructing the pelvic outlet. A manual disimpaction was accomplished under intermittent fetal monitoring, but during the procedure a sudden continuous fetal bradycardia was detected. This required an emergency Cesarean section under general anesthesia, which occurred with no intraoperative complications. Eleven minutes later, a female neonate weighing 2900g was delivered, with a one-minute Apgar score of three and a five-minute score of five. Neonatal resuscitation was required. There were no anatomic abnormalities of the umbilical cord and placenta and a normal cord insertion to the placenta was present. The newborn baby was admitted to our Neonatal Intensive Unit Care until the 26^th^ day of life for neonatal asphyxia, metabolic acidosis and seizures. A cerebral MRI revealed severe hypoxic–ischemic lesions and after discharge, the baby was referred to a cerebral palsy specialized center.

**Figure 2:**
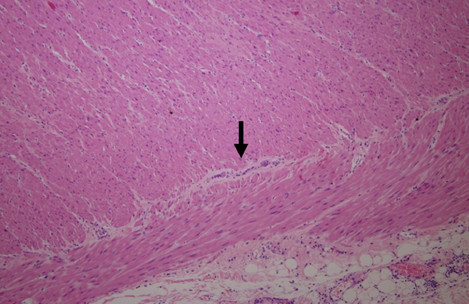
Histopathological finding of hypoganglionosis showing a markedly decreased number of nerve cells (arrow) in the myenteric plexus (hematoxylin and eosin × 100).

After delivery, the mother had a rapid recovery with reestablishment of normal bowel function 24 hours after the surgery. After being discharged, she started attending regular gastroenterological appointments. She maintained mild constipation that was easily controlled with magnesium hydroxide, but one year after delivery she attended our hospital with chronic constipation unresponsive to medical therapy. An MRI performed at that time revealed persistent dilatation of her colon and we decided to perform a rectosigmoid resection, which occurred without complications. A histopathological analysis revealed a significant reduction of nerve cells in the myenteric and submucous plexus and hypertrophy of the muscularis mucosa (Figures 2). The acetylcholinesterase activity was not evaluated because this test is not available in our institution.

## Discussion

Megacolon refers to a dilatation of the colon that is not caused by mechanical obstruction [5]. It is the radiological finding of a large group of congenital or acquired diseases characterized by an intestinal innervation defect and currently known as intestinal innervation disorders or dysganglionosis. According to the widely accepted standardized nomenclature of a multidisciplinary consensus conference held in 1990 [6], dysganglionosis can be classified into five types:

Agenetic type:

Classic Hirschsprung’s disease

Long-segment aganglionosis

Total aganglionosis of the colon

Total intestinal aganglionosis

Ultrashort-segment disease

Hypogenetic type:

Isolated hypoganglionosis

Hypogenesis of nerve cells of the myenteric and submucous plexus

Dysgenetic type:

Intestinal neuronal dysplasia A

Intestinal neuronal dysplasia B

Acquired conditions:

Chagas’ disease (infection with *Trypanosoma cruzi*)

Other neurological, systemic and metabolic diseases

Medication induced conditions

Combined forms:

Hirschsprung’s disease + intestinal neuronal dysplasia B

Hirschsprung’s disease + hypoganglionosis

Hypoganglionosis + intestinal neuronal dysplasia B

Hypogenetic nerve cells in the submucous plexus + intestinal neuronal dysplasia B

IH, a hypogenetic type of dysganglionosis, is one of the rarest subtypes of intestinal innervation disorders, accounting for only 5% of all cases [7,8]. Although clinically resembling classic Hirschsprung’s disease (HD), with chronic refractory constipation as one of the most common symptoms [9] and an overall male-to-female ratio of 3:1 [8], the median age at diagnosis is significantly higher in patients with IH than those with HD (90.5% of cases are diagnosed in the newborn period) and can be made as late as the age of 17 years [8]. The diagnosis of IH is histological and requires the demonstration of the following histological characteristics in three to five full-thickness biopsies of the intestinal wall: very low or absent activity of acetylcholinesterase activity in the mucosa; significant reduction of nerve cells in the myenteric and submucous plexus; and hypertrophy of the muscularis mucosa and muscle layers [7,8]. Since conservative therapies cannot usually alleviate symptoms effectively, the treatment of choice for IH involves resection of the affected bowel segment [8,10].

Being a rare condition, IH is even more rarely diagnosed during pregnancy. Until now, there have been only eight studies [1-4,11-14] published regarding megacolon diagnosis during pregnancy, most of them published more than 20 years ago. In fact, these studies do not provide enough evidence to establish accurate recommendations on the management of megacolon during pregnancy.

There are several issues regarding the management of dysganglionosis during pregnancy that have to be considered. From an obstetric point of view, there are two major concerns. One is the need to consider more rigorous prenatal care as it is not known if these diseases pose a greater risk of obstetric complications, such as preterm labor, preterm premature rupture of membranes and fetal growth restriction. The other concern regards the timing and type of delivery. Should the women be delivered sooner? Should they have an elective Cesarean section or should we encourage a normal delivery? To try to answer these questions, we should look to the previews reports in the literature (Table 1). In 1955, Grasby and Higgins published a report of three cases of megacolon diagnosed during pregnancy [1]. All three women delivered by forceps extraction and in all cases a dystocia occurred caused by fecal impaction, requiring the manual removal of the fecaloma. In 1979, Resta *et al*. [3] published another case of a 26-year-old woman with a previous diagnosis of HD, who had an uneventful pregnancy and delivery. In 1982, Hjortrup *et al*. [4] reported the first case of HD diagnosis during pregnancy. Their patient presented at 33 weeks with an acute bowel obstruction. She underwent an emergency laparotomy with a temporary sigmoidostomy and Cesarean section. The most recent published article [2] reports the case of an idiopathic megarectum diagnosed at 28 weeks of gestation, in a patient with a previous history of severe chronic constipation unresponsive to medical therapy. An aggressive polyethylene glycol regimen allowed their patient to carry the pregnancy to term (although she had several episodes of severe constipation requiring manual disimpaction) and to have a normal vaginal delivery.

**Table 1:** Summary of published studies regarding megacolon management during pregnancy

**Authors (year)**	**Number of patients**	**Gestational age at diagnosis (weeks)**	**Treatment and complications during pregnancy**	**Gestational age at delivery (weeks)**	**Mode of delivery**	**Birth weight (g)**	**Apgar score**	**Obstetric complications**	**Maternal and neonatal outcome**
Grasby and Higgins (1955) [1]	3	16	Laxative (senna) No complications	36-40	Forceps extraction	NR	NR	Dystocia caused by fecal impaction; manual removal of the fecaloma	Good
Resta *et al*. (1979) [3]	1	Diagnosis previous pregnancy	None	38	Vacuum extractor	3200	8/9	None	Good
Hjortrup *et al*. (1982) [4]	1	33	Temporary sigmoidostomy (because of acute bowel obstruction)	33	Cesarean section	1500	NR	None	Good
Grossmann *et al*. (2000) [2]	1	28	Laxative (polyethylene glycol); several episodes of severe constipation requiring manual disimpaction	Term	Normal vaginal delivery	NR	NR	None	Proctocolectomy three months postpartum

NR: not referred.

We believe from our own and others’ experience that in these cases of megacolon it is very important to anticipate the possibility of dystocia. Although these women should not necessarily deliver sooner, if there is a fecaloma obstructing the pelvic outlet during labor, the mode of delivery must be reconsidered. One can choose to have a Cesarean section, because manual disimpaction can take a long time and endanger the well-being of the fetus. In our case, a sudden fetal bradycardia was detected during manual disimpaction. It is possible that this procedure induced severe uterine contractions that may have compromised the uteroplacental circulation. Nevertheless, we know that Cesarean section increases the risk of maternal morbidity and mortality and an additional risk for future pregnancies, such as placenta previa. So, should a vaginal delivery be considered? Perhaps yes, although it is very important to continuously monitor the fetus when performing manual disimpaction. This measure may have prevented the poor outcome in our case.

From a gastroenterological point of view, there are also some concerns that must be considered. It is important to remember that some diagnostic imaging procedures should be avoided during pregnancy. According to the American College of Obstetricians and Gynecologists, ultrasonography and MRI are not associated with known adverse fetal effects and can be used during pregnancy [15]. However, the current guidelines of the Food and Drug Administration require labeling MRI devices to indicate that the safety of MRI with respect to the fetus ‘has not been established’. Although most studies evaluating MRI safety during pregnancy show no ill effects, it is good practice to only perform MRI during pregnancy if the potential benefit justifies the potential risk to the fetus, avoiding this imaging examination particularly during the first trimester. Another important issue concerns the medical and surgical treatment of these conditions during pregnancy. In pregnant women, dysganglionosis should be first managed with medical treatment alone. Conservative measures include a high-fiber, high-water intake; exercise; the use of enemas, bulking agents and laxatives; and manual disimpaction. The best laxatives are osmotic agents (magnesium salts, lactulose, sorbitol or polyethylene glycol) that are secure during pregnancy. Stimulant laxatives (senna, bisacodyl, cascara sagrada) should be used as a last resort because they may induce deterioration in the ability of the colon to evacuate, although safe in pregnant women. When medical therapies fail, surgical measures involving resection of the affected bowel segment should be considered.

## Conclusion

We report a very rare case of IH first diagnosed during early pregnancy. Although a pregnancy can be carried out normally in a patient with these rare intestinal innervation diseases, further investigations are mandatory to delineate guidelines for clinical management of dysganglionosis during pregnancy.

## Consent

Written informed consent was obtained from the patient for publication of this case report and accompanying images. A copy of the written consent is available for review by the Editor-in-Chief of this journal.

## Abbreviations

HD: Hirschsprung’s disease; IH: isolated hypoganglionosis; MRI: magnetic resonance imaging.

## Competing interests

The authors declare that they have no competing interests.

## Authors’ contributions

AF wrote the manuscript and was responsible for the literature research. IM, FP, MA and CB were involved in critically revising the manuscript for important intellectual content. All authors have read and approved the final manuscript.
